# Foetal Macrosomia and Foetal-Maternal Outcomes at Birth

**DOI:** 10.1155/2018/4790136

**Published:** 2018-08-08

**Authors:** Sahruh Turkmen, Simona Johansson, Marju Dahmoun

**Affiliations:** ^1^Department of Clinical Sciences, Obstetrics and Gynecology, Sundsvalls Research Unit, Umeå University, Umeå 90185, Sweden; ^2^Department of Obstetrics and Gynecology, Sundsvall County Hospital, Sundsvall 85186, Sweden

## Abstract

To investigate how macrosomia affects foetal-maternal birth outcomes, we conducted a retrospective cohort study of singleton pregnant women who gave birth at gestational age ≥37+0 weeks. The patients were divided into three groups according to birth weight: “macrosomia” group, ≥4500 g, n=285; “upper-normal” group, 3500–4499 g, n=593; and “normal” group, 2500–3499 g, n=495. Foetal-maternal and delivery outcomes were compared among the three groups after adjustment for confounders. Caesarean section was more frequent in the macrosomia group than in upper-normal and normal groups. The duration of labour (p < 0.05) and postpartum care at the hospital (p < 0.001) were the highest in the macrosomia group. Increased birth weight was associated with higher risks of shoulder dystocia (p < 0.001), increased bleeding volume (p < 0.001), and perineal tear (p < 0.05). The Apgar score at 5 minutes (p < 0.05), arterial cord pH (p < 0.001), and partial pressure of O2 (p < 0.05) were lower, while the arterial cord partial pressure of CO2 was higher (p < 0.001), in the macrosomia group. Macrosomia has potentially serious impacts for neonate and mother as a result of a complicated and occasionally traumatic delivery.

## 1. Introduction

Macrosomia is a term used to describe an estimated foetal weight or birthweight of more than 4500 g, but a birthweight above 4000 g is also commonly used to define this condition. The term is often used as a synonym for large-for-gestational-age foetuses (birthweight >90^th^ percentile), and nearly 10% of all pregnancies are affected by macrosomia [1, 2]. The factors associated with this condition include a history of macrosomia, multiparity, maternal obesity prior to conception, excessive weight gain during pregnancy, advanced gestational age, and maternal diabetes as the strongest risk factor; however, in many high-birthweight cases, the cause is unknown [3, 4]. Earlier studies have shown that macrosomia can increase the risk of unfavourable delivery outcomes, including instrumental and/or caesarean deliveries, postpartum haemorrhage, shoulder dystocia, collarbone fracture, brachial plexus injury, and asphyxia [5–7]. Some authors have suggested that the complications during delivery caused by macrosomia can be prevented by delivery via elective caesarean section [8]. This strategy is considered justified only when the estimated foetal weight is over 4500 g in women with diabetes or over 5000 g in women without diabetes [9]. Another strategy to overcome the negative impacts of macrosomia is early induction of labour to reduce the likelihood of foetal growth; however, the increased risks of maternal and neonatal morbidity and mortality associated with induction should be taken into consideration [10, 11]. Several studies have suggested that labour induction is associated with an increased risk of caesarean section delivery, with no reduction in the number of birth-related injuries [12–14]. A recent randomized control study suggested that labour induction for macrosomic foetuses at gestational age 37–39 weeks reduces the risks of dystocia and collarbone fracture while increasing the likelihood of spontaneous vaginal birth [15].

Although accurate estimation of birthweight prior to labour and identification of foetuses at risk are challenges, there are no existing common guidelines as to how to manage macrosomia. In this retrospective study, we attempted to determine the effects of birth weight on labour, foetal-maternal outcomes, and obstetric complications. The purpose of this study is to increase the knowledge and nursing care preparedness of the emergency obstetric staff in managing macrosomia.

## 2. Materials and Methods

A retrospective cohort study was undertaken of all singleton pregnant women who gave birth at gestational age ≥37 weeks + 0 days in the maternity unit of a county hospital in Sundsvall, Sweden, over a 5-year period (from January 1, 2011 to December 31, 2015). The patients were divided into three groups according to foetal birth weight: ≥4500 g (macrosomia group), 3500–4499 g (upper-normal group), and 2500–3499 g (normal group). The maternal and foetal outcomes were evaluated and compared among the three groups.

The aim of the study was to evaluate the associations between birthweight and foetal-maternal outcomes. The patients who met the criteria detailed below were identified by searching our hospital medical records using Obstetrix (Siemens Corporation, Upplands Väsby, Sweden), a Swedish electronic medical record system that is specialised for prenatal care and childbirth. In Obstetrix, the pregnancy is followed in a logical and structured manner, from enrolment in the prenatal health care centre to arrival to the maternity unit and the time of delivery. This study was approved by the Regional Ethical Review Board of Umeå, Sweden.

Patients with a singleton pregnancy who gave birth at gestational age ≥37 weeks + 0 days to a foetus with a birthweight ≥2500 g were included in the study. Since the risk of morbidity for newborn and women increases drastically, when the birth weight is more than 4500 g [16], macrosomia was defined as a birthweight >4500 g. Patients were excluded for the following reasons: a multiple pregnancy, infection or contagious disease, history of psychiatric care, more than one delivery by caesarean section, or premature birth (before gestational age 37 weeks). Maternal hypothyroidism and asthma were not considered exclusion criteria.

Maternal demographic characteristics (age, body mass index [BMI], parity, previous caesarean section, and systemic disease) and the following outcomes were assessed: time from the start of delivery (cervical dilation ≥4 cm) to birth, period of postpartum care at the hospital (time from delivery to discharge), delivery method, shoulder dystocia, genital tract injury (vaginal or perineal rupture), anal sphincter injury, and bleeding volume at birth.

The following foetal outcomes were assessed: neonatal complications attributed to macrosomia in terms of the Apgar score at 5 minutes and umbilical cord arterial blood parameters (pH, partial pressure of O2 [pO2], partial pressure of CO2 [pCO2], and a base excess [BE]).

*Statistical Analysis*. All statistical analyses were performed using Statistical Package for Social Sciences version 23 (SPSS Inc., Chicago, IL, USA). Descriptive statistics were used to present the data, which were divided into categorical, ordinal, and continuous variables. The normality of the distribution of the data was tested using Shapiro–Wilks test. Continuous nonparametric variables were evaluated by the Kruskal–Wallis and Mann–Whitney U tests and presented as medians (range), while categorical/ordinal variables were evaluated by chi-square test. The relationships between variables were determined by adjusting for confounders in multiple logistic regression analyses. Stepwise linear regression analyses were performed for continuous variables and binary logistic regression analyses for categorical variables.

## 3. Results

**Figure 1:**
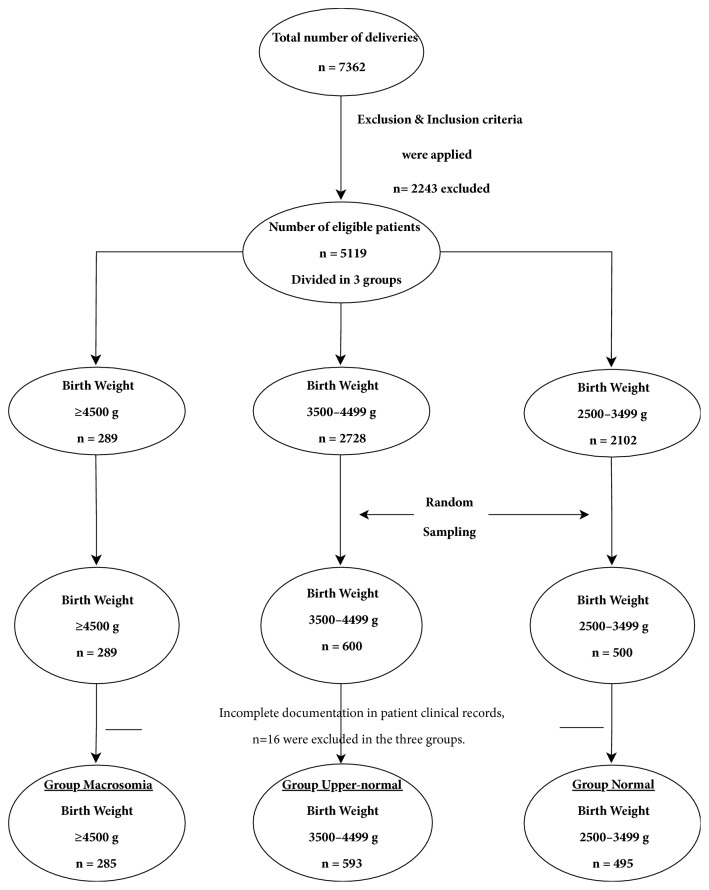
Flowchart. A visual representation of the sequence of steps and decisions made to include patients.

A total of 7362 women delivered at the maternity unit of a county hospital in Sundsvall, Sweden, from January 1, 2011 to December 31, 2015. The patients' medical records were checked, and those with incomplete data records were excluded. After applying the inclusion and exclusion criteria and randomization, 1373 women were included in the study (Figure 1). The patients were divided into three groups according to birth weight: macrosomia group, ≥4500 g (n = 285); upper-normal group, 3500–4499 g (n = 593); and normal group, 2500–3499 g (n = 495). As the sample sizes of groups were highly unequal and the number of macrosomic neonates differed substantially from the numbers of newborns in the other two groups, to preclude a general loss of statistical power, we reduced the number of patients in upper-normal and normal groups using simple randomization. The randomization was performed in the Excel program (Microsoft® Office, 2013), by providing an arbitrary number from 0 to 1 in both upper-normal and normal groups. After sorting the patients in ascending order in one and each group, the first 600 respective 500 patients were selected in upper-normal and normal groups, respectively, and included in study. The number of patients in macrosomia group remained unchanged. 16 patients with incomplete documentation in clinical records were excluded afterwards from the three groups, and the number of patients who were analyzed decreased to 1373.

Overall differences were found among the three groups in terms of maternal age (p < 0.001), gestational age at birth (p = 0.001), maternal BMI (p < 0.001), and diabetes during pregnancy (p < 0.01); however, the number of previous caesarean section deliveries did not differ among the groups (Table 1). The mode of starting delivery (p = 0.001), delivery method (p = 0.001), labour duration (p = 0.013), length of postpartum care at the hospital (p < 0.001), bleeding volume (p < 0.001), and number of shoulder dystocia events (p < 0.001) were also different among the groups (Table 2). Neonatal outcomes, including the Apgar score at 5 minutes (p = 0.001), arterial cord pH (p < 0.001), arterial cord pO2 (p = 0.002), and arterial cord pCO2 (p < 0.001), also showed differences among the groups (Table 3).

In comparisons of macrosomia and upper-normal groups, women in macrosomia group had a greater BMI (p < 0.001), gestational age at birth (p < 0.001), and bleeding volume (p < 0.001) and a longer labour duration (p = 0.004) and postpartum care period (p < 0.001). The neonates of macrosomia group had a higher umbilical cord pCO2 (p < 0.001) but a lower pO2 (p < 0.001), Apgar score at 5 minutes (p = 0.044), and arterial pH (p = 0.017). The results suggest that the neonates were more stressed in macrosomia group than in upper-normal group during labour. Maternal age and umbilical cord arterial BE were not different between macrosomia and upper-normal groups (Tables 2 and 3).

Comparison between macrosomia and normal groups showed significant differences suggestive of a complicated labour for both the mothers and neonates of macrosomia group. Maternal age (p < 0.001), gestational age at birth (p < 0.001), maternal BMI (p < 0.001), and bleeding volume (p < 0.001) were greater and the labour duration (p = 0.017) and postpartum care period (p < 0.001) longer in macrosomia group than in normal group. Regarding the neonatal outcomes, the Apgar score at 5 minutes (p = 0.001), pO2 (p = 0.007), and umbilical cord arterial pH (p < 0.001) were lower, and pCO2 (p < 0.001) was higher in macrosomia group than in normal group. Umbilical artery BE was not different between the groups.

Comparisons of upper-normal and normal groups revealed that the patients in upper-normal group had a significantly greater maternal age (p = 0.004), gestational age at birth (p < 0.001), maternal BMI (p < 0.001), and bleeding volume (p = 0.001) and a longer postpartum care period (p = 0.005). Cord arterial pH (p = 0.049) and pCO2 (p = 0.018) were also different between the groups (lower pH and higher pCO2 level in upper-normal group), suggesting more stress among neonates in upper-normal group; however, the Apgar score at 5 minutes, arterial cord pO2, BE, and duration of delivery were not different between the two groups (Tables 2 and 3).

To compare the observed and expected data among the three groups, we performed chi-square tests with Bonferroni correction of the p-values. Among the women in macrosomia group, diabetes (x^2^ = 20.801, df = 2, p < 0.001) was more frequent, delivery was started mainly by induction (x^2^ = 23.286, df = 4, p < 0.001), and caesarean section was the most common delivery method (x^2^ = 41.155, df = 6, p < 0.001). Shoulder dystocia (x^2^ = 15.805, df = 2, p < 0.001) and perineal tear (x^2^ = 13.727, df = 6, p = 0.033) were also more frequent in this group. However, the numbers of cervical, vaginal, and anal sphincter tears and previous caesarean section deliveries were not significantly different among the groups. Comparisons of two groups showed differences between macrosomia and upper-normal groups in terms of the frequencies of diabetes (x^2^ = 8.348, df = 1, p = 0.004), shoulder dystocia (x^2^ = 6.502, df = 1, p = 0.016), and perineal tear (x^2^ = 8.461, df = 3, p = 0.037) and in the mode of starting delivery (x^2^ = 21.242, df = 2, p < 0.001) and the delivery method (x^2^ = 16.858, df = 3, p = 0.001). Macrosomia and normal groups differed in terms of the mode of starting delivery (x^2^ = 11.214, df = 3, p = 0.011) and delivery method (x^2^ = 11.214, df = 3, p = 0.011). Upper-normal and normal groups differed in the frequencies of diabetes (x^2^ = 17.733, df = 1, p < 0.001) and shoulder dystocia (x^2^ = 12.268, df = 1, p < 0.001), mode of starting delivery (x^2^ = 14.165, df = 2, p = 0.001), and delivery method (x^2^ = 32.702, df = 3, p < 0.001) (Table 2).

Multiple logistic regression analyses were performed to identify correlations and confounders, and the results were shown in Table 4. The analysis revealed that birth weight was associated with diabetes (p < 0.005) and positively correlated with maternal BMI (p ≤ 0.001), gestational age at birth (p ≤ 0.001), and maternal age (p = 0.020). The total bleeding volume at birth was positively correlated with birth weight, delivery method (vaginal deliveries being associated with the least and caesarean section with the most bleeding), and vaginal and cervical tears. The total bleeding volume at birth was also affected by the mode of starting delivery; bleeding volume was the highest for caesarean section and induction (both p < 0.001). The duration of labour was positively correlated with maternal age and was associated with the mode of starting delivery and delivery method (p < 0.05). The period of postpartum care at the hospital appeared to be influenced by the delivery method, which increased with instrumental and caesarean section deliveries; the mode of starting delivery (hospitalization time was shorter when delivery started spontaneously); and by labour duration, gestational age at birth, bleeding volume, the grades of vaginal and anal sphincter tears, and diabetes (hospitalization time was twice as long for women with diabetes) (p < 0.05). Birth weight (p = 0.003) and diabetes (p = 0.048) were predictor of shoulder dystocia. The Apgar score at 5 minutes was negatively associated with shoulder dystocia, maternal BMI, vaginal tears, delivery method, and diabetes (p < 0.05). The cord arterial pH was negatively associated with the hospitalization stay after birth, birth weight, vaginal tearing, total bleeding volume, and delivery method (p < 0.05). The cord pO2 was correlated with the delivery method, diabetes, and vaginal tearing (p < 0.05), while the cord pCO2 value was correlated with maternal BMI, birth weight, and diabetes (p < 0.05).

## 4. Discussion

The results of this study suggest that macrosomia is associated with increased risks of caesarean section and trauma to the birth canal and the foetus. Advanced maternal age and gestational age at birth, high BMI, and the presence of diabetes emerged as predisposing factors for macrosomia. As the rate of caesarean section delivery was higher among women with macrosomic foetuses, birth weight may influence the delivery method. The number of patients with grade 4 perineal tear, as a labour-associated injury, was also higher in this group. Macrosomia can increase the rate of shoulder dystocia and maternal bleeding volume at birth.

The risk of morbidity for newborn and women increases drastically when the birth weight is more than 4500 g [1, 4, 17]. In another trial, the authors studied birth weight categories to determine predictive thresholds of adverse outcomes. The study results suggested that a definition of macrosomia as >4000 g may be useful for the identification of increased risks of labour and newborn complications, >4500 g may be more predictive of neonatal morbidity, and >5000 g may be a better indicator of infant mortality risk [2]. Our results confirm partly their findings that adverse outcomes such as labour complications, delivery method, mode of delivery start, and neonatal morbidity differ across varying birth weight thresholds, but we have not observed any difference in rate of neonatal mortality between weight categories, and moreover, many outcomes were similar in two groups with birth weight <4500 g (see Tables 2 and 3). Differences between two studies may be explained by the fact that the sample size in our study was smaller and the study conducted in a geographically limited population.

In the macrosomia group, the incidence of spontaneous delivery was lower, but the rates of elective caesarean section and labour induction were higher, compared with the other two groups. These results are in accordance with other studies showing that the rate of caesarean section delivery was increased among women who delivered macrosomic foetuses after labour induction [5, 18, 19]. Surprisingly, we found that normal group (the lowest birthweight neonates) had the highest rate of instrumental delivery via vacuum. Although the rate of complications during labour in nonmacrosomic neonates is not generally expected to be high, it is probable that conditions other than stress that required delivery assistance or acceleration (e.g., a prolonged second phase of delivery) were more common in this group. Foetal distress and/or the threat of asphyxia were rare in this group, as indicated by the higher Apgar score at 5 minutes and cord arterial pH in group 3 than in the other two groups.

Some studies have suggested that macrosomia is associated with a higher rate of injuries during labour [4, 5]. Although the number of patients with perineal injuries was low in all groups of our study, there was an overall difference among the three groups (see Table 2). There were twice as many patients with anal sphincter tears in the macrosomic group compared with the other two groups. An earlier study suggested a positive association between cervical and/or vaginal lacerations and macrosomia [19]; however, our results showed that the number of vaginal tears in the macrosomia group was less than half those in the other two groups, and the number of cervical tears did not differ among groups. An explanation for this may be that macrosomic neonates are commonly delivered via caesarean section, whereas normal vaginal and instrumental deliveries are more frequent in the other two groups.

Earlier studies have shown that macrosomia increases the risk of shoulder dystocia [15, 18, 20]. Some studies have suggested that offspring born to women with diabetes are at higher risk of experiencing shoulder dystocia, but this risk was decreased when labour was induced at 38–39 weeks of gestation [20–22]. Our finding that the risk of shoulder dystocia was higher among macrosomic neonates is consistent with the above-mentioned studies, but we did not find an association between diabetes and shoulder dystocia. We found that a higher foetal weight was associated with the presence of diabetes and advanced gestational age; other studies have also suggested an increased prevalence of large-for-gestational-age neonates in women with diabetes [21]. The inconsistency regarding the association between diabetes and shoulder dystocia may result from differences in study populations and/or policies regarding the management of pregnancy in diabetic women, as labour may be induced at an earlier gestational age in women with diabetes. Prophylactic caesarean delivery may also be considered for suspected macrosomia with an estimated foetal weight of at least 4500 g in women with diabetes and at least 5000 g in women without diabetes, but the clinical effectiveness of a prophylactic caesarean delivery is controversial [23–25]. Analysis of our data shows that, in the group of women with diabetes, the delivery method has no effect on the Apgar score at 5 min and the risk of shoulder dystocia (p > 0.05, OR 0.133), although the results need to be interpreted cautiously, due to low number of diabetes patients in our study.

We evaluated the impact of macrosomia on foetal outcomes. Macrosomic neonates had a lower arterial cord pH, pO2, and Apgar score at 5 minutes and a higher pCO2 compared with the neonates of the other groups. These parameters are accepted indicators of the vitality and wellbeing of newborns. We found that instrumental/surgical deliveries and diabetes are negatively associated with the Apgar score at 5 minutes. Moreover, the number of shoulder dystocia cases among macrosomic neonates was higher, and the Apgar score at 5 minutes of the shoulder dystocia cases was lower. Lower Apgar scores are seen more frequently in macrosomic neonates when the delivery is complicated by shoulder dystocia [26], although other studies were unable to demonstrate any difference in the Apgar score between normal-birthweight and macrosomic neonates [27]. According to our findings, macrosomia seems to increase the risk of foetal impairment, and the low Apgar score at 5 minutes in the macrosomic group may be a consequence of the complications associated with macrosomia.

We also evaluated the impact of birthweight on delivery outcomes. The postpartum care period in the hospital was longer in women with diabetes and/or macrosomic neonates. This finding can be explained by the high frequencies of caesarean section deliveries and perineal tears in this group. It has been suggested that labour induction for foetal macrosomia can increase the risk of complications such as genital tract injuries, thereby increasing the hospital stay for recovery of these women [18]. In addition, the risk of foetal-maternal complications increases in women with diabetes. The postpartum control of diabetes is a very important issue for the well-being of the mother and newborn because of the increased incidence of hypoglycaemia, indicating a need for close monitoring and prolonged care in the hospital.

We identified four variables (maternal age, maternal BMI prior to pregnancy, gestational age at delivery, and maternal diabetes) as potential predisposing factors and predictors of macrosomia. Many previous studies have also suggested that diabetes is the strongest predisposing factor for macrosomia [28, 29]. It has also been shown that women with diabetes are more likely to be obese and to gain more weight during pregnancy; furthermore, maternal BMI is a predictor of diabetes and is therefore considered a risk factor for macrosomia [30, 31]. Diabetes during pregnancy elevates the mother's blood glucose and insulin levels, causing insulin to circulate from the mother to baby, which can lead to excessive fat deposits and macrosomia. Our findings are in line with those of other studies that showed positive correlations among maternal BMI, maternal diabetes, and foetal macrosomia. Because the weight of the foetus increases with gestational age, it is not unusual that foetal macrosomia is associated with a higher gestational age. In addition, we found a positive association between increased maternal age and macrosomia, although a previous study showed no such effect [28].

In conclusion, macrosomia may place the mother and neonate at risk for adverse outcomes. Our findings suggest that the delivery of a macrosomic neonate has potentially serious impacts for neonates and mothers in terms of a difficult and occasionally traumatic delivery. We identified maternal BMI, maternal age, gestational age at birth, and maternal diabetes as risk factors that influence the development of macrosomia in pregnant women. Furthermore, an earlier induction of labour in pregnant women with presumed foetal macrosomia may reduce the risks of caesarean section and trauma to the birth canal and foetus.

However, because earlier trials to investigate the negative effects of macrosomia have shown contradictory results, more studies are needed to determine a safe and effective method for proper management of macrosomia. The purpose of this study was to increase the knowledge and nursing care preparedness of the emergency obstetric staff in managing macrosomia. The results of this study are of course not decisive, but it can still illustrate circumstances to perceive the complications and situations that may occur during the delivery of a foetus with macrosomia.

## Figures and Tables

**Table 1:** Demographic and baseline characteristics of the patients.

	G Macrosomia (n = 285)	G Upper-normal (n = 593)	G Normal (n = 495)	G comparisons
Maternal age (years)	30 (26)	30 (33)	29 (33)	b, c, c
BMI (kg/m^2^)	26.8 (53.5)	24.5 (54.9)	23.5 (41.2)	a, b, c
Gestational age (weeks)	41 (5)	40 (6)	39 (5)	a, b, c
Previous caesarean, n (%)	26 (9.1%)	51 (8.6%)	38 (7.7%)	

Data are presented as numbers (%) or medians (range). G, group; a, p < 0.01 for G1 versus G2; b, p < 0.01 for G1 versus G3; c, p < 0,01 for G 2 versus G3; and n, number of patients.

**Table 2:** Maternal and delivery outcomes.

	G Macrosomia (n = 285)	G Upper-normal (n = 593)	G Normal (n = 495)	G comparisons
Diabetes, n (%)	12 (4.2%)	7 (1.2%)	1 (0.2%)	a, b
Labour duration (h)	6 (84)	5 (101)	5 (109)	a, b
Postpartum care (h)	49 (306)	18 (162)	26 (221)	a, b, c
Bleeding at birth (mL)	400 (2700)	300 (2450)	300 (2750)	a, b, c
Shoulder dystocia	7 (2.5%)	3 (0.5%)	0 (0%)	a, b
Tearing				
Vaginal	17 (6%)	32 (5.4%	32 (6.5)	
Cervical	1 (0.4%)	1 (0.2%)	1 (0.2%)	
Perineal				a
Grade II	7 (2.5%)	17 (2.9%)	18 (3.6%)	
Grade III	10 (3.5%)	21 (3.5%)	11 (2.2%)	
Grade IV	4 (1.4%)	0 (0%)	1 (0.2%)	
Anal sphincter				
< half	5 (1.8%)	13 (2.2%)	7 (1.4%)	
> half	2 (0.7%)	4 (0.7%)	2 (0.4%)	
Total	7 (2.5%)	3 (0.5%)	3 (0.6%)	
Delivery start				a, b
Spontaneous	185 (64.9%)	467 (78.8%)	382 (77.2%)	
Induction	74 (26%)	83 (14%)	79 (16%)	
Caesarean	26 (9.1%)	43 (7.3%)	34 (6.9%)	
Delivery method				a, b, c
Normal vaginal	190 (66.7%)	456 (76.9%)	369 (74.5%)	
Forceps	0 (0%)	1 (0.2%)	1 (0.2%)	
Vacuum	15 (5.3%)	39 (6.6%)	60 (12%)	
Caesarean	80 (28.1%)	97 (16.4%)	65 (13.1%)	

Mann–Whitney U, Kruskal–Wallis, and chi-square tests were used for the statistical analyses. Data are presented as the number (%) or the median (range). G, group; a, p < 0.05 for G1 versus G2; b, p < 0.01 for G1 versus G3; c, p < 0,01 for G 2 versus G3; and n, number of patients.

**Table 3:** Neonatal outcomes.

	G Macrosomia (n = 285)	G Upper-normal (n = 593)	G Normal (n = 495)	G comparisons
Birthweight (g)	4674 (4568 to 4840 g)	3816 (3657 to 4050)	3210 (3032 to 3358 g)	a, b, c
Apgar score at 5 min	10 (8)	10 (10)	10 (7)	a, b
Umbilical artery pH	7.2 (0.5)	7.2 (0.6)	7.2 (0.4)	a, b, c
pO2	2.6 (7.2)	3 (17.5)	3 (6.4)	a, b
pCO2	8.1 (12.5)	7.5 (14.2)	7.2 (9.2)	a, b, c
BE	−4.7 (23.8)	−5.2 (26.2)	−5.3 (19.9)	

Mann–Whitney U and Kruskal–Wallis tests were used for the statistical analyses. Data are presented as the number (%) or the median (range). Variation in birthweight showed by interquartile range (Q3-Q1). G, group; a, p < 0.05 for G1 versus G2; b, p < 0.01 for G1 versus G3; c, p < 0,05 for G 2 versus G3; n, number of patients; pO2, partial pressure of O2; pCO2, partial pressure of CO2; and BE, base excess.

**Table 4:** Regression analysis.

	Dependent variable						
	Birth weight OR (95% CI)	Shoulder dystocia OR (95% CI)	Anal Sphinct. tears OR (95% CI)	Duration of Labour (B)	Bleeding Volume (B)	Apgar 5 min (B)	PPPC (B)
Age (years)	1.0 (0.9-1.0) *∗*	0.9 (0.8 – 1.1)	1.0 (0.9 – 1.0)	(0.2) *∗∗∗*	(-2.6)	(0.01)	(-0.6)
BMI (kg/m2)	0.9 (0.9 – 1.0) *∗∗*	0.9 (0.8 – 1.1)	1.0 (0.9 – 1.0)	(0.1)	(1.4)	(0.01) *∗*	(0.2)
Diabetes (yes/no)	10.2 (3.5 - 29.4) *∗∗*	26.5 (1.0 - 684) *∗*	0.7 (0.8 – 5.1)	(-6.8)	(-16.7)	(0.4) *∗*	(8.1) *∗*
Gestational age at birth (week)	0.5 (0.4-0.6) *∗∗∗*	2.1 (0.7 – 6.0)	1.1 (0.9 – 1.4)	(-0.2)	(1.2)	(0.01)	(-2.2) *∗∗*
Birth weight (g)	-	1.0 (0.9 - 1.0) *∗∗*	1.0 (1.0 - 1.1) *∗*	(-0.01)	(0.1) *∗*	(-0.6)	(-0.01)
Apgar score at 5 min	-	0.3 (0.1 – 0.6)	0.9 (0.7 – 1.2)	(0.2)	(5.0)	-	(-2.0)
Shoulder dystocia	-	-	7.5 (1.5 - 36.3) *∗*	(-2.2)	(-31.3)	(1.7) *∗∗∗*	(14.3)
Delivery method	-	0.7 (0.2 – 2.0)	0.9 (0.7 – 1.1)	(2.1) *∗∗∗*	(52.2) *∗∗∗*	(0.9) *∗∗∗*	(8.1) *∗∗∗*
Mode of starting labour	-	1.8 (0.6 – 5.7)	1.1 (0.8 – 1.6)	(7.1) *∗∗∗*	(37.3) *∗∗∗*	(0.01)	(6.0) *∗∗∗*
Vaginal tears	-	13 (2.2 - 78.5) *∗∗*	37.2 (18.6 - 74.3) *∗∗∗*	(1.7)	(146.7) *∗∗*	(0.2) *∗∗*	(11.9) *∗*
Cervical tears	-	0.01 (0.01 – 0.1)	0.4 (0.2 – 0.9)	(-4.6)	(503.5) *∗∗*	(0.1)	(-0.7)
Anal Sphincter tears	-	9.7 (0.5 – 178)	-	(0.3)	(65.1)	(0.2)	(10.3) *∗*
Bleeding volume (ml)	-	1.0 (0.9 – 1.0)	0.9 (0.3 – 2.6)	(-0.01)	-	(0.01)	(0.01) *∗∗∗*
Duration of labour (h)	-	0.9 (0.8 – 1.0)	1.0 (0.9 – 1.0)	-	(-0.9)	(0.01)	(0.7) *∗∗∗*
Postpartum care at the hospital (h)	-	1.0 (0.9 – 1.0)	1.0 (1.0 – 1.1)	(0.05) *∗∗*	(1.5)	(-0.01)	-

OR, odds ratio; CI, confidence interval; B, B-coefficient; and PPPC, period of postpartum care at hospital. *∗* = p < 0.05; *∗∗* = p < 0.01; *∗∗∗* = p < 0.001.

## Data Availability

The data used to support the findings of this study are available from the corresponding author upon request.
